# Editorial: Human-centered AI at work: common ground in theories and methods

**DOI:** 10.3389/frai.2024.1411795

**Published:** 2024-04-17

**Authors:** Annette Kluge, Uta Wilkens, Verena Nitsch, Corinna Peifer

**Affiliations:** ^1^Organizational and Business Psychology, Ruhr University Bochum, Bochum, Germany; ^2^Institute of Work Science, Ruhr University Bochum, Bochum, Germany; ^3^Institute of Industrial Engineering and Ergonomics, RWTH Aachen University, Aachen, North Rhine-Westphalia, Germany; ^4^Department of Psychology, University of Lübeck, Lübeck, Germany

**Keywords:** human-AI-teaming, configurational theory, worker participation, work design, social systems, augmentation, team-centered, HRM

Human-centered AI at work is being theorized, investigated, and developed in various disciplines providing different definitions and interpretations. Involved disciplines range from information science, machine learning, engineering and robotics, medicine up to ergonomics/work science, psychology, sociology, pedagogics, philosophy, business studies, law and labor relations, just to mention the core disciplines involved in the current debate.

The state-of-the-art presented in the Research Topic's contributions includes lessons learned from socio- technical system design, group work and humane working conditions, negative short-term and long-term consequences in working with automation, design principles of human-autonomy-teaming and effective collaboration between humans collaborating with humans in face of technology, human-machine interaction, workplace democracy and configurational theory.

Authors contribute with reviews, disciplinary and interdisciplinary theory outlines, empirical analysis for tool assessment as well as outcome measures and case illustration. In addition, they provide visionary perspectives to guide future research. The Research Topic includes contributions which (1) systematize the state-of-the-art discourses and methods, (2) specify the operationalization of variables and their relationships, and (3) outline a vision for future practice and related research.

This forms a basis for the development of a research agenda in human-centered AI at work.

## State-of-the-art discourses and methods

Berretta et al. conduct a scoping review for a research network analysis and identify five dominant clusters in the field of human-AI-teaming (HAIT) facing (1) human variables, (2) task-dependent variables, (3) AI explainability, (4) AI-driven robotic systems, and (5) effects of AI performance on human perception. It becomes obvious that current research streams are dominated by techno-centric and engineering perspectives but might define a starting point for further elaborating on more human-centric approaches as supported by the authors. They emphasize communication and collaboration requirements in sharing intents, situational awareness and shared mental models as well as trust among the team members as an issue of HAIT.

Buschmeyer et al. specify the state-of-the-art in the development of methods that are aligned with ISO norms in human-centered design and propose to transfer this framework to AI-based work systems. They introduce a validated instrument assessing (1) system characteristics that are particularly important from the users' perspective; (2) work-related characteristics with respect to mental load and augmentation potential, and (3) cross-task work characteristics. These criteria and the underlying validation define a starting point for future method development.

A research design for measuring the effects of AI tools on human cognitive performance is introduced by Wallinheimo et al.. The authors present a pre-post-measurement among language professionals applying a tool for 5 weeks within a test design. Positive effects for the individual are identified in particular with respect to working memory.

## Operationalization of variables and their relationships

Wilkens et al. conduct a cross-disciplinary systematic literature review for specifying criteria as operational benchmarks for human-centered AI at work. In total, they explore eight criteria of human-centricity, (1) trustworthiness and (2) explainability face challenges of technology development, (3) prevention of job loss, (4) health, and (5) human agency & augmentation face challenges of employee development, and (6) compensation of systems' weaknesses, (7) integration of user-domain knowledge, (8) accountability & safety culture reflect challenges of organizational development. With reference to configurational theory the authors argue that different criteria matter in different contexts and depending on stakeholders' responsibility.

Haipeter et al. also contribute toward the contextualization of AI-related research in this field. They refer to the discourse of German speaking sociologists and stress the positive moderator impact of employees' participation in AI implementation as an issue of accountability. The authors illustrate their theoretical argument with a case study description from the German telecommunication industry in which work councils participated in the development of a responsible AI declaration.

Bocklisch and Huchler add further criteria of successful AI implementation for the context of AI-based team settings. Their review among writings from sociology specifies (1) complementarity, (2) shared knowledge & goals, and (3) bounded autonomy as a prerequisite to gain (4) human and team trust in implemented AI.

Mazarakis et al. present a draft for a comprehensive cross-disciplinary model with respect to outcome factors. They pled for the integration of expertise of human factors engineering, human computer interaction, psychology, information science, and adult education in order to envision a future in which AI systems and humans collaborate synergistically to gain higher levels of productivity, innovation, participation and wellbeing.

## Vision for future research and practice

Hagemann et al. illustrate hybrid multi-team systems in which human-centered AI emphasize the need for team-centeredness that aligns goals, communication, and decision making with humans. They outline the requirements for such future work contexts with team-centered AI from a sociotechnical perspective, such as cognitive competence, reinforcement learning, and semantic communication.

Fenwick et al. describe the lack of human considerations in HRM tech design and thus develop a vision for the future role of HRM in face of human-AI work systems. They specify the technical, human, and ethical challenges of future HRM systems fully-embedded in a human-centered approach. In this context, they define human-centric AI as AI tools that prioritize and enhance the human experience by making them more intuitive, empathetic, and aligned with human values and needs.

## Is there a common ground?

It became clear that a pure focus on technology is too narrow for human-centered approaches but that an exclusive focus on individual variables is also too narrow.

These writings underline that there is a range of criteria indicating human-centered AI at work. The selection of these criteria for empirical analysis varies between disciplines and use fields. It becomes obvious that overall frameworks and criteria exist but need to be adapted to the concrete context as unit of analysis and stakeholders involved whether it is e.g., technology development, human-AI team building or bargaining between status groups.

Hence, it seems that the work system and job characteristics, but especially the team focus and interaction with and around AI which matter as a future unit of analysis. Established methods and leading communities and their impact become clear by the help of these articles.

The Research Topic's contributions show that different perspectives co-exist and—to increase complexity- they co-exist on different levels: individual workplace, team, and organization. On the organizational level Haipeter et al., Wilkens et al., and Fenwick et al. address organizational and social practices of human-centered AI. The team level is addressed in contributions by Hagemann et al., Bocklisch and Huchler, and Berretta et al.. On the workplace level Wallinheimo et al., Mazarakis et al., and Buschmeyer et al. discuss aspects and measurable criteria for designing human-centered workplaces, jobs and AI-assisted tasks.

Hence, based on the Research Topic's contributions, we propose that the common ground of human-centered AI at work

- is embedded in the social systems of an organization, including organizational practices such as HR processes, production processes, and participation processes;
- is value driven- by the striving for decent working conditions (e.g., SDG #8),
- but goes beyond the demand for decent work and sketches images of the augmented worker, working with intelligent systems that fulfills the requirements of social belonging and relatedness and self-actualization and development;
- acknowledges employees as social beings with needs regarding social contact and motives related to other social beings (“teaming”) in the organization;
- augments human capabilities without imposing additional load due to “bad design” in direct human-AI-interaction, and while performing a work task;
- can be assessed and evaluated by means of subjective and objective measures

The Research Topic's visionary contributions underline that human-centered AI needs a focus on interrelated systems to evaluate whether ethical criteria are fulfilled and what are the outcomes and effects on different levels. A set of criteria and variables that needs to be adapted to the use case and unit of analysis were specified. In this way, the research contributions together provide a common ground in human-centered AI at work.

## Author contributions

AK: Conceptualization, Writing – original draft, Writing – review & editing. UW: Conceptualization, Writing – original draft, Writing – review & editing. VN: Writing – review & editing. CP: Writing – review & editing.

